# Supplementary material for: Cryo-electron microscopy of IgM-VAR2CSA complex reveals IgM inhibits binding of Plasmodium falciparum to Chondroitin Sulfate A
Source: Nat Commun. 2023 Oct 12;14:6391. doi: 10.1038/s41467-023-41838-x (PMC10570280; doi:10.1038/s41467-023-41838-x)
Supplement: Supplementary file 1 — Supplementary Information [file 41467_2023_41838_MOESM1_ESM.pdf]

Cryo-electron microscopy of IgM-VAR2CSA complex reveals IgM inhibits binding of *Plasmodium falciparum* to Chondroitin Sulfate A

Reetesh Raj Akhouri[1,3]*, Suchi Goel[2#], Ulf Skoglund[1#]

*Materials and Correspondence

akhourirr@gmail.com

Senior Author

[1] Okinawa Institute of Science and Technology Graduate University, Japan

[2] Indian Institute of Science Education and Research Tirupati, India

[3] Present address

Indian Institute of Technology Madras, Chennai, India

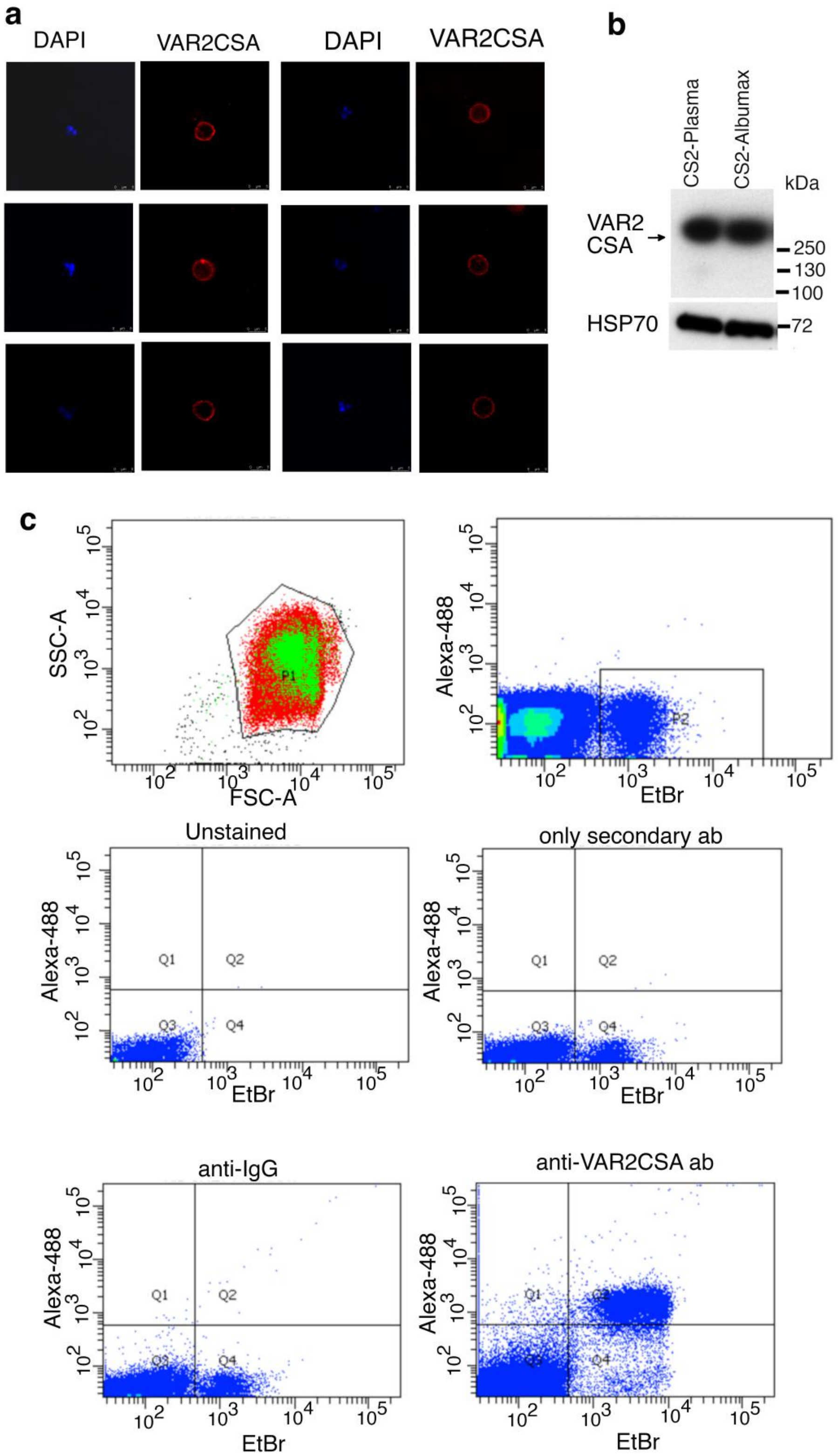

a
DAPI
VAR2CSA
DAPI
VAR2CSA
b
CS2-Plasma
CS2-Albumax
kDa
VAR2
CSA
250
130
100
HSP70
72
c
SSC-A
FSC-A
P1
Alexa-488
EtBr
P2
Unstained
only secondary ab
anti-IgG
anti-VAR2CSA ab
Q1
Q2
Q3
Q4

Supplementary Fig. 1. a Representative images of three experiments of VAR2CSA staining on IEs of CS2 using anti-VAR2CSA antibodies visualized using confocal microscope with LASX software at 63X objective lens. Scale bar-5µm**. b** Representative image of three experiments of western blot using anti-VAR2CSA and anti-PfHSP70 antibodies comparing the expression of VAR2CSA in CS2 grown in media containing normal plasma and albumax with HSP70 as loading control. **c** FACS analysis of CS2 IEs using anti-VAR2CSA antibodies. The RBCs were gated using FSC and SSC plots from the unstained cultures (P1 gate, top left) and IEs were gated after EtBr staining (top right, P2 gate). For analysis of staining, 10,000 IEs were counted and analyzed for alexa fluor 488 fluorescence after staining with anti-VAR2CSA antibodies, pre-immune rabbit IgG alone followed by anti-rabbit IgG alexa fluor 488, only secondary antibody were used as controls. Q1 represents population positive for only alexa fluor 488 signal, Q2 positive for both alexa fluor 488 and EtBr fluorescence, Q3 for unstained population and Q4 positive for only EtBr fluorescence.VAR2CSA expressing IEs are present in Q2 area.

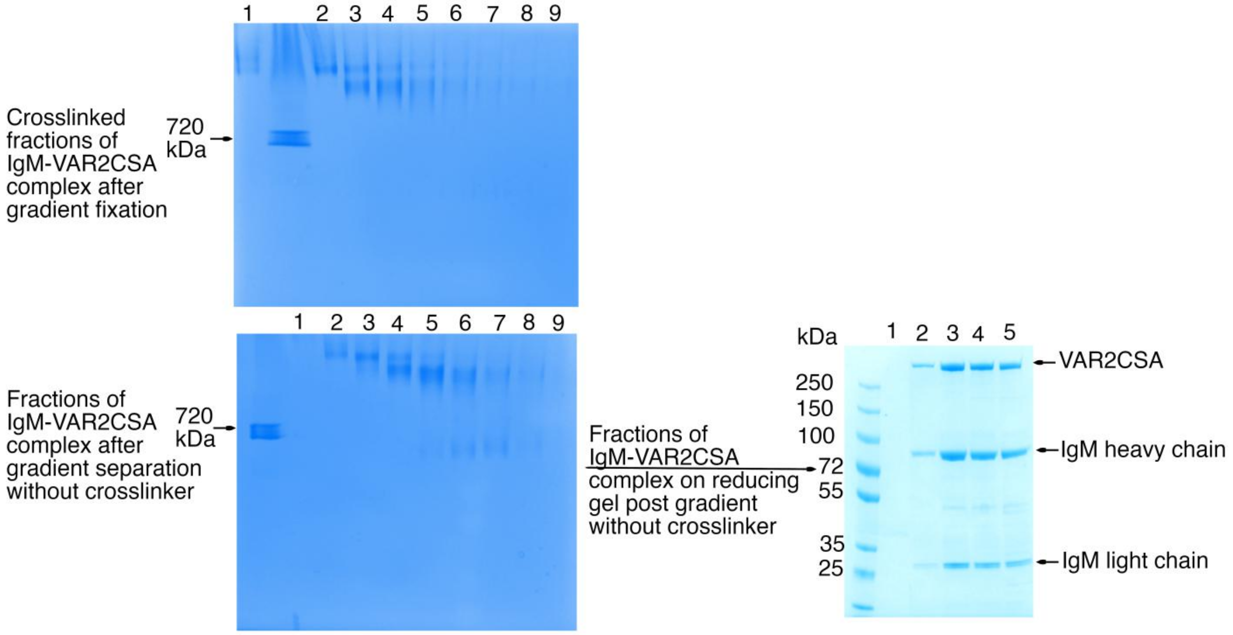

Supplementary Fig. 2. Fractions of gradient fixation (GraFix) of IgM-VAR2CSA complex in presence of crosslinker (top) and absence of crosslinker (bottom) analyzed after ultracentrifugation and fractionation on Native-PAGE. The non-crosslinked fractions 1 to 5 were also resolved on reducing SDS-PAGE(bottom, right) to show the presence of VAR2CSA as well as IgM in the complex.

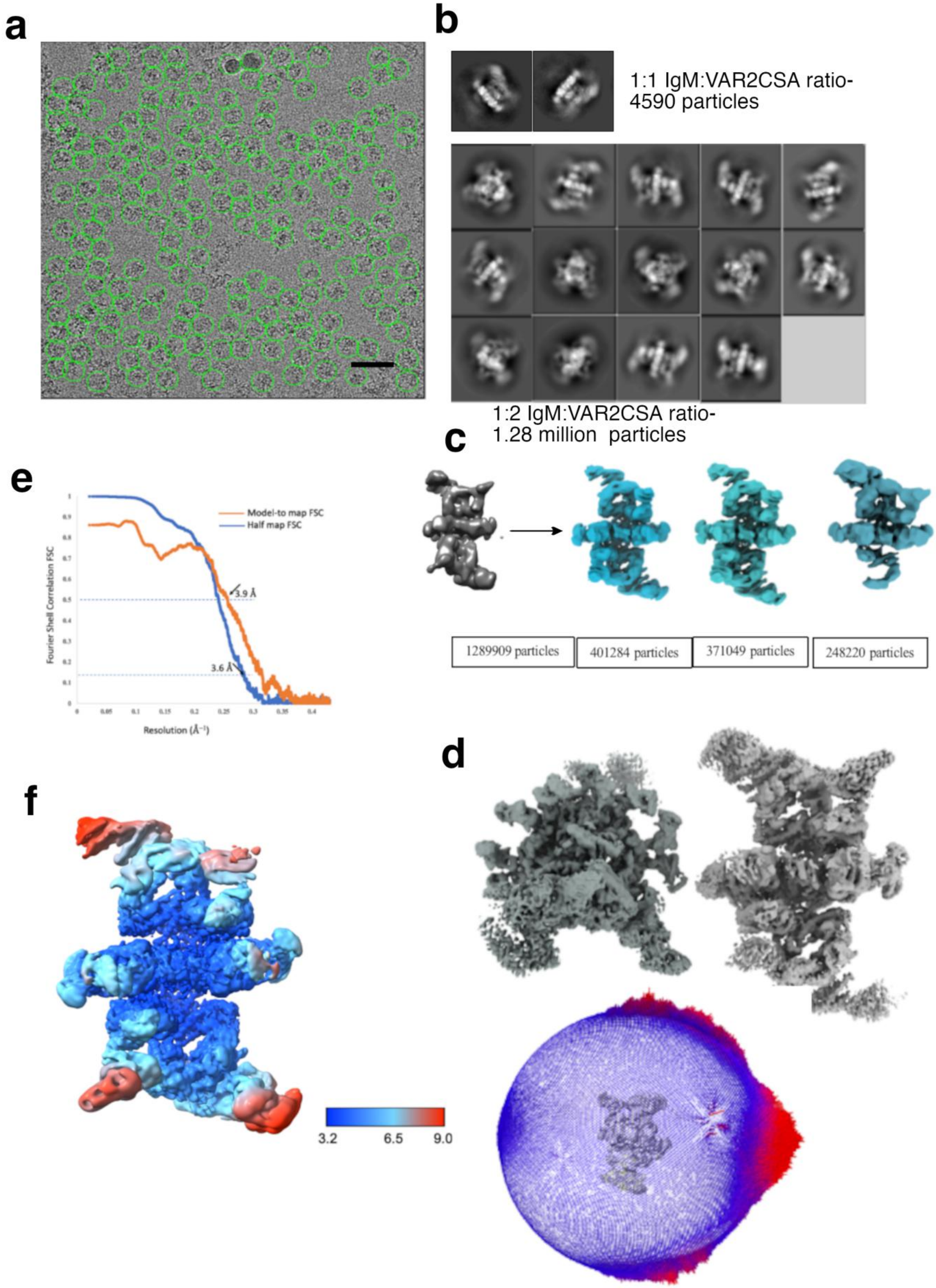
a
b
1:1 IgM:VAR2CSA ratio-
4590 particles
1:2 IgM:VAR2CSA ratio-
1.28 million particles
c
1289909 particles
401284 particles
371049 particles
248220 particles
e
Model-to map FSC
Half map FSC
3.9 Å
3.6 Å
Fourier Shell Correlation FSC
Resolution (Å⁻¹)
d
f
3.2
6.5
9.0

Supplementary Fig. 3. Workflow for Cryo-EM image collection, processing and structure determination. **a** Representative raw micrograph of IgM-VAR2CSA complex collected on 300KeV Titan Krios microscope using falcon3 camera. The green circles shows picked particles. **b** 2D class averages of IgM-VAR2CSA complex. **c** Initial model generated from the 2D-class averages. 3D class averages along with number of particles in each class averages. **d** 3D-Autorefined cryo-EM map and Eulerian angle distribution of particles in angular build. **e** Gold standard Fourier Shell Correlation (FSC) of the half maps (blue) and of model-to-map FSC (orange). **f** Local resolution filtered map of IgM-VAR2CSA complex using RELION Localres.

a

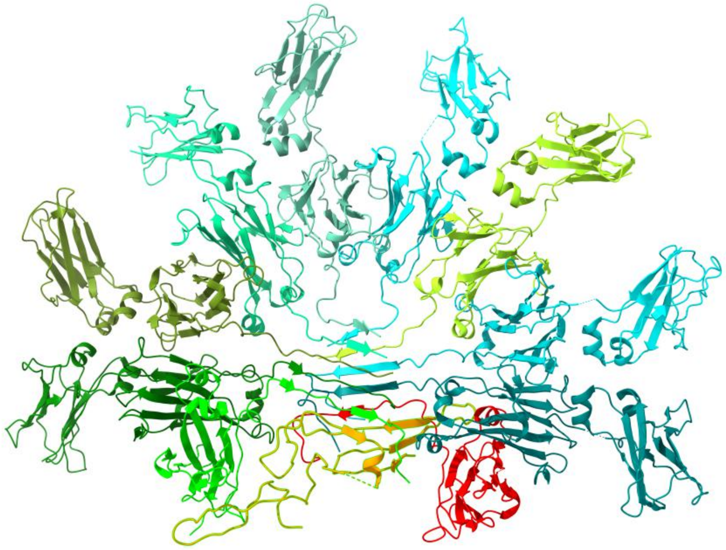

b

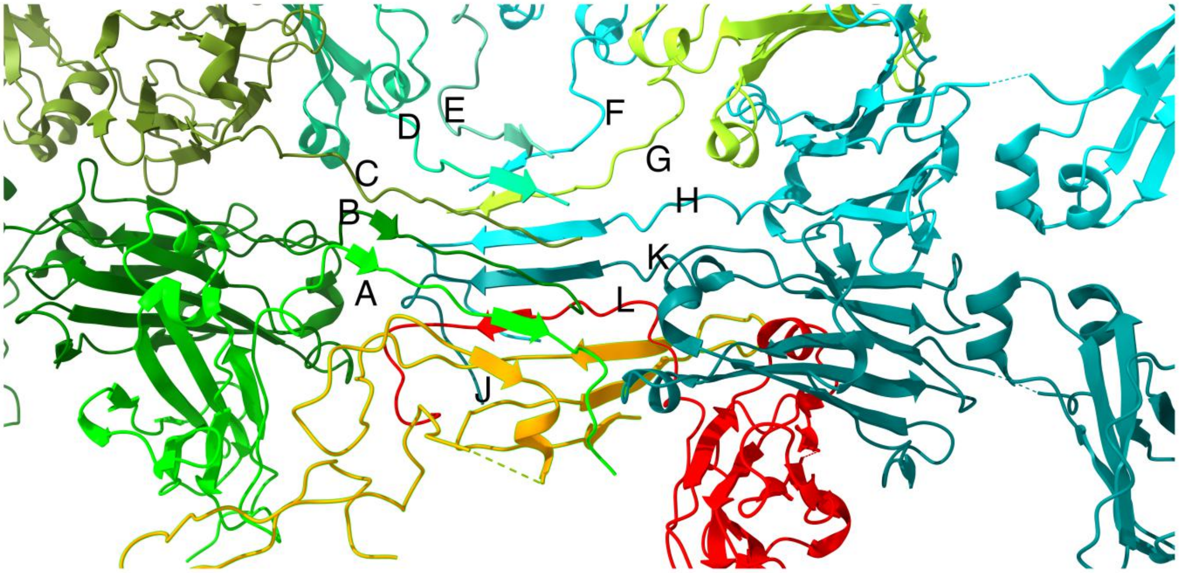

Supplementary Fig. 4. a The overall organization of IgM tailpieces depicted in different colors **b** Five parallel β-sheets of A-E and F-L tailpieces; A (lime), B (green), C (olive drab), D(spring green), E(aquamarine), F (Cyan), G(green yellow),H(dark turquoise) and K(Teal), L(red) and J (orange) stack anti-parallel to stabilize the IgM core and facilitate formation of two identical faces.

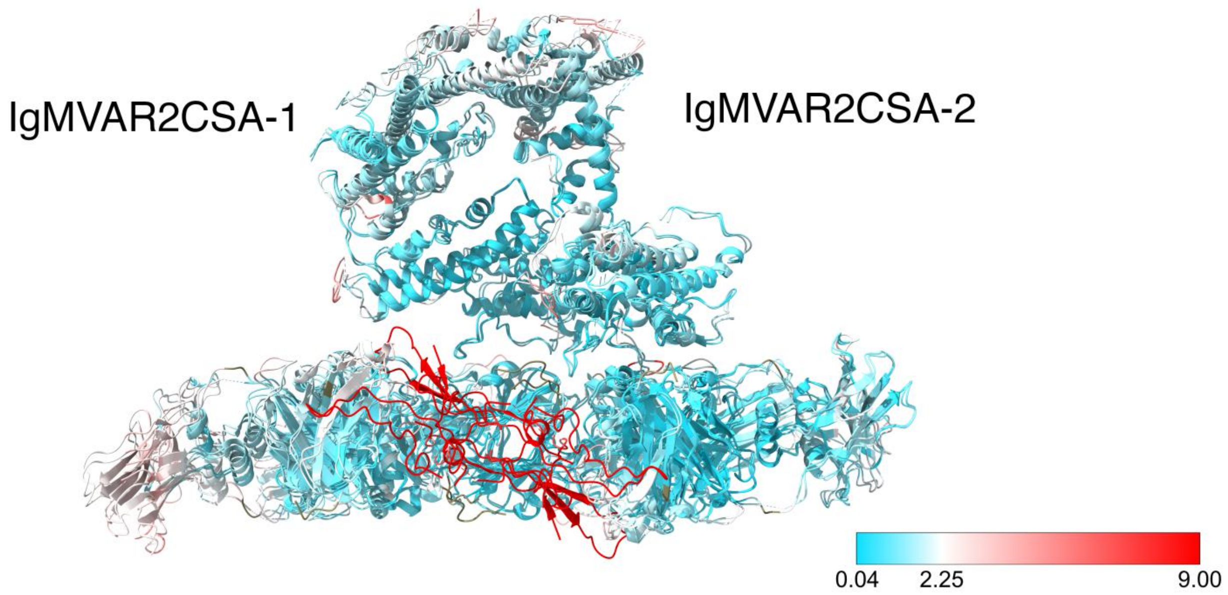

Supplementary Fig. 5. Sequence RMSD based comparison of IgMVAR2CSA-1 and IgMVAR2CSA-2. The gradient from cyan to red show the increasing RMSD. The RMSD for 2696 residues is 0.99 Å and across 3261 residues, RMSD is 6.98 Å, due to J chain.

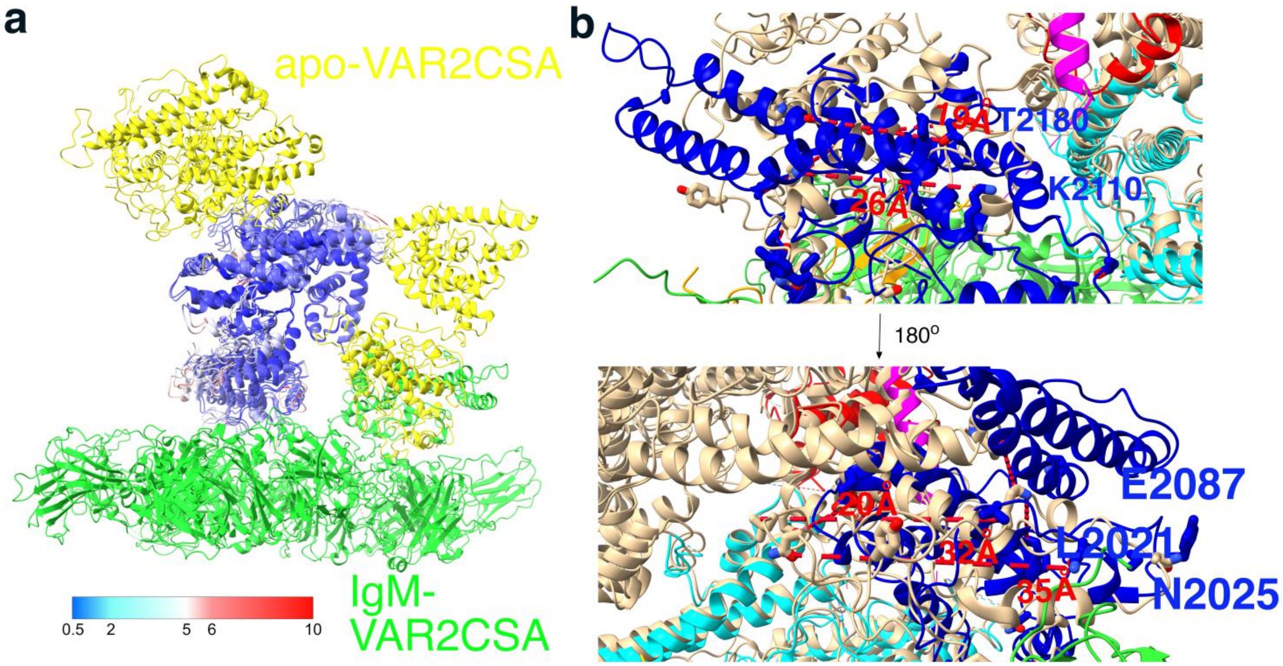

Supplementary Fig. 6. Comparison of apo-VAR2CSA(pdb7B52+pdb7NNH) and VAR2CSA in the complex. **a** Sequence RMSD based comparison of apo-VAR2CSA(yellow) and IgM-VAR2CSA (green). The gradient from blue to red show the increasing RMSD. The RMSD for 580 residues is 1.36 Å and across 865 residues, RMSD is 2.070 Å. Due to large conformational shift, Matchmaker did not include DBL5ε. **b** apo-VAR2CSA (beige). For the complex, IgM is colored green, VAR2CSA domains from Fig. 2b: ID2- red, DBL3x-cyan, ID3-magenta, DBL4ε-brown, DBL5ε-blue. The right panel shows shift of DBL5ε domain upon binding to IgM. The dashed red line shows the distances between corresponding residues of DBL5ε in apo-VAR2CSA and IgM-VAR2CSA.

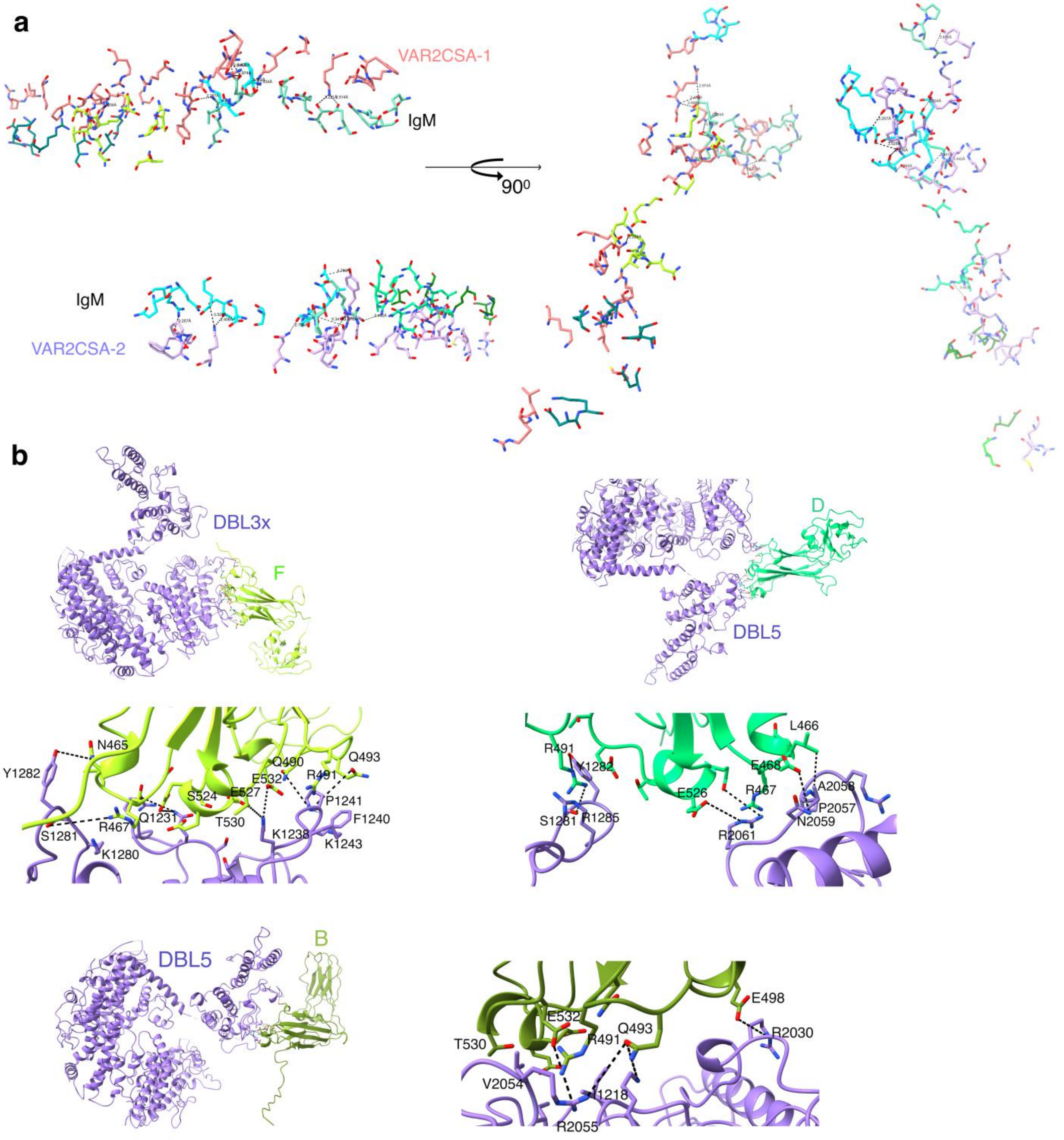

Supplementary Fig. 7. a The orthogonal views showing the interfacing residues of VAR2CSA and IgM in the complex where 2 VAR2CSA (VAR2CSA-1; salmon, VAR2CSA-2 (medium purple) are bound by different chains of an IgM **b** Interacting interfaces along with respective atomic view of the interfacing residues between chain M (VAR2CSA) and chain F, chain D and chain B of IgM.

```
                          1        10        20        30        40
PfDd2_120005600/1-338     KCNENASKDTKINKSETSSKCSEPIYIRGCQSKRYDGFISPGK
PfKH02_120006100/1-339    KCKENESTNNKMKSSETSSKCSEPIYIRGCQSKTYDGFISPGK
PfCD01_120005700/1-326    KCKENESTDTKINKSETSSKCNKTNYIRGCQSKRYDGKIFPGK
PfSN01_120006700/1-341    KCNENESTDTKINKSETSCDLNATNYIRGCQSKTYDGKIFPGK
PfML01_120006200/1-329    KCNENASKNTKINPSVISSDCSEPIYIRGCQSKTYDGFISPGK
PfSD01_120005900/1-339    KCKENASKNTKINKSGKSCDCNEPIYIRGCQSKTYDGFISPGK
PF3D7_1200600/1-319       KCKENESTNNKMKSSETSCDCSEPIYIRGCQPKIYDGKIFPGK
PfNF54_120005700/1-319    KCKENESTNNKMKSSETSCDCSEPIYIRGCQPKIYDGKIFPGK
PfHB3_120006000/1-342     QCKEIQNTDTKMKSSETSSKCNEPIYIRGCQPKRYDGFISPGK
PfIT_120006100/1-328      KCKENESTDTNINKSETSCDLNATNYIRGCQSKTYDGKIFPGK
Pf7G8_120005700/1-341     KCKENESTDTKMKSSETSCDLNATNYIRGCQSKTYDGKIFPGK
PfKH01_000031900/1-324    ..KEIQNTDTNINKSGKSCDLNATNYIRGCQSKTYDGKIFPGK
PfGA01_120005700/1-325    KCNENESTNNKMKSSETSCDCSEPIYIRGCQSKTYDGKIFPGK
PfGB4_120005700/1-326     KCNENASKNTKINKSETSSDCSEPIYIRGCQSKTYDGFISPGK
PfKE01_120006100/1-325    KCNENESTDTKINKSETSSDCSEPIYIRGCQSKTYDGFISPGK

                               50        60        70        80
PfDd2_120005600/1-338     GGEKQWICKDTIIHGDTNGACIPPRTQNLCVGELWDKRYGGRS
PfKH02_120006100/1-339    GGEKQWICKDTITHGDTNGACIPPRTQNLCVGNLWDKSYGGRS
PfCD01_120005700/1-326    GGEKQWICKDTITHGDTNGACIPPRTQNLCVGELWDKSYGGRS
PfSN01_120006700/1-341    GGEKQWICKDTIIHGDTNGACIPPRTQNLCVGELWDKRYGGRS
PfML01_120006200/1-329    GGEKQWICKDTITHGDTNGACIPPRTQNLCVGNLWDKSYGGRS
PfSD01_120005900/1-339    GGEKKWICKDTITHGDTNGACIPPRTQNLCVGELWDKRYGGRS
PF3D7_1200600/1-319       GGEKQWICKDTIIHGDTNGACIPPRTQNLCVGELWDKRYGGRS
PfNF54_120005700/1-319    GGEKQWICKDTIIHGDTNGACIPPRTQNLCVGELWDKRYGGRS
PfHB3_120006000/1-342     GGEKQWICKDTIIHGDTNGACIPPRTQNLCVGNLWDKRYGGRS
PfIT_120006100/1-328      GGEKQWICKDTIIHGDTNGACIPPRTQNLCVGELWDKSYGGRS
Pf7G8_120005700/1-341     GGEKQWICKDTIIHGDTNGACIPPRTQNLCVGNLWDKSYGGRS
PfKH01_000031900/1-324    GGEKQWICKDTIIHGDTNGACIPPRTQNLCVGNLWDKSYGGRS
PfGA01_120005700/1-325    GGEKQWICKDTITHGDTNGACIPPRTQNLCVGELWDKRYGGRS
PfGB4_120005700/1-326     GGEKKWICKDTITHGDTNGACIPPRTQNLCVGELWDKSYGGRS
PfKE01_120006100/1-325    GGEKKWICKDTITHGDTNGACIPPRTQNLCVGELWDKRYGGRS

                            90        100       110       120
PfDd2_120005600/1-338     NIKNDTKESLKNKLKNAIQKETELLYEYHDTGTAIISRNPMKE
PfKH02_120006100/1-339    NIKNDTKESLKNKLKNAIQKETELLYEYHDKGTAIISRNPMKE
PfCD01_120005700/1-326    NIKNDTKELLKNKLKNAIQKETELLYEYHDKGTAIISQNDKKG
PfSN01_120006700/1-341    NIKNDTKESLKNKLKNAIQKETELLYEYHDKGTATISRNPMKE
PfML01_120006200/1-329    NIKNDTKESLKNKLKNAIQKETELLYEYHDKGTATISRNPMKE
PfSD01_120005900/1-339    NIKNDTKESLKNKLKNAIQKETELLYEYHDKGTAIISRNPMKE
PF3D7_1200600/1-319       NIKNDTKESLKQKIKNAIQKETELLYEYHDKGTAIISRNPMKG
PfNF54_120005700/1-319    NIKNDTKESLKQKIKNAIQKETELLYEYHDKGTAIISRNPMKG
PfHB3_120006000/1-342     NIKNDTKESLKNKLKNAIQKETELLYEYHDKGTAIISRNPMKE
PfIT_120006100/1-328      NIKNDTKELLKEKIKNAIHKETELLYEYHDTGTAIISKNDKK.
Pf7G8_120005700/1-341     NIKNDTKESLKNKLKNAIQKETELLYEYHDKGTAIISRNPMKE
PfKH01_000031900/1-324    NIKNDTKESLKNKLKNAIQKETELLYEYHDKGTAIISQNDKK.
PfGA01_120005700/1-325    NIKNDTKESLKNKLKNAIQKETELLYEYHDKGTAIISQNDKK.
PfGB4_120005700/1-326     NIKNDTKESLKNKLKNAIQKETELLYEYHDKGTAIISQNDKK.
PfKE01_120006100/1-325    NIKNDTKESLKNKLKNAIQKETELLYEYHDKGTAIISQNDKK.
```

```
                               130       140       150       160       170
                                 .         .         .         .         .
PfDd2_120005600/1-338       GGEDGKGKQKEGGEEAN..NNFNGLPKGFCHAVQRSFIDYKNM
PfKH02_120006100/1-339      GGEDGKGKQKEGGEEAN..NNSNGLPKGFCHAVQRSFIDYKNM
PfCD01_120005700/1-326      Q...............KGKNDPNGLPKGFCHAVQRSFIDYKNM
PfSN01_120006700/1-341      GGEDGKGKQKEGGEKANNNKNSNGLPKGFCHAVQRSFIDYKNM
PfML01_120006200/1-329      GGEDGKGKQKEGGEKANN.NNSNGLPKGFCHAVQRSFIDYKNM
PfSD01_120005900/1-339      GGEDGKGKQKEGGEEAN..NNSNGLPKGFCHAVQRSFIDYKNM
PF3D7_1200600/1-319         QKE.........KEEKN..NDSNGLPKGFCHAVQRSFIDYKNM
PfNF54_120005700/1-319      QKE.........KEEKN..NDSNGLPKGFCHAVQRSFIDYKNM
PfHB3_120006000/1-342       GGEDGKGKQKEGGEEAN..NNSNGLPKGFCHAVQRSFIDYKNM
PfIT_120006100/1-328        ............GQKGK..NDPNGLPKGFCHAVQRSFIDYKNM
Pf7G8_120005700/1-341       GGEDGKGKQKEGGEEAN..NNSNGLPKGFCHAVQRSFIDYKNM
PfKH01_000031900/1-324      ............GEKGK..NDPNGLPKGFCHAVQRSFIDYKNM
PfGA01_120005700/1-325      ............EKANN..NNSNGLPKGFCHAVQRSFIDYKNM
PfGB4_120005700/1-326       ............EKANN..NNSNGLPKGFCHAVQRSFIDYKNM
PfKE01_120006100/1-325      ............EKANN..NNSNGLPKGFCHAVQRSFIDYKNM
```

```
                                   180       190        200       210
                                     .         .          .         .
PfDd2_120005600/1-338       ILGTSVSTYEYIGKLQEDIKKIIEQGR.KPK..DKTVGSGADK
PfKH02_120006100/1-339      ILGTSVNIYEYIGKLQEDIKKIIEQETTKQN..GKTVGSGADK
PfCD01_120005700/1-326      ILGTSVNTYEYIGKLQEDIKKIIEKGTTKQN..GKTVGSGADK
PfSN01_120006700/1-341      ILGTSVNIYEYIGKLQEDIKKIIEKGTTKQN..GKTVGSGADK
PfML01_120006200/1-329      ILGTSVNIYEYIGKLQEDIKKIIEQETPQQN..GKTVGSGADK
PfSD01_120005900/1-339      ILGTSVNIYEYIGKLQEDIKKIIEKGTPQQN..GKTVGSGADK
PF3D7_1200600/1-319         ILGTSVNIYEYIGKLQEDIKKIIEKGTTKQN..GKTVGSGAEN
PfNF54_120005700/1-319      ILGTSVNIYEYIGKLQEDIKKIIEKGTTKQN..GKTVGSGAEN
PfHB3_120006000/1-342       ILGTSVNIYEYIGKLQEDIKKIIEKGTPQQKDKIGGVGSGADK
PfIT_120006100/1-328        ILGTSVNIYEHIGKLQEDIKKIIEKGTPQQKDKIGGVGSSTEN
Pf7G8_120005700/1-341       ILGTSVNTYEYIGKLQEDIKKIIEKGTPQQKDKIGGVGSSTEN
PfKH01_000031900/1-324      ILGTSVNIYEYIGKLQEDIKKIIEQERTKTK..EKRVGSGAEN
PfGA01_120005700/1-325      ILGTSVNIYEHIGKLQEDIKKIIEQETTKQN..GKTVG.GADK
PfGB4_120005700/1-326       ILGTSVNIYEHIGKLQEDIKKIIEQERTKTK..EKTVGSGADK
PfKE01_120006100/1-325      ILGTSVNTYEYIGKLQEDIKKIIEQGR.KPK..EKRVGSGADK
```

```
                                   220       230        240       250
                                     .         .          .         .
PfDd2_120005600/1-338       VNDWWKEIEKDMWGAVKCGIKTIKKQKK.NGTFNGDECGVSPP
PfKH02_120006100/1-339      VNDWWKEIEKDMWGAVKCGIKTIKKQKK.NGTYTGNECGVSPP
PfCD01_120005700/1-326      VNDWWKEIEKDMWGAVKSGIKTIKKQ.NNKCTYTGNECGVSPP
PfSN01_120006700/1-341      VNDWWKEIEKDMWGAVKCGIKTIKKQ.NNKCTYTGDECGIFPP
PfML01_120006200/1-329      VNDWWKEIEKDMWGAVKCGITKINKKQKKNGTYTGDECGVSPP
PfSD01_120005900/1-339      VNDWWKEIEKDMWGAVKCAITKINKKNN.KCTYTGNECGVSPP
PF3D7_1200600/1-319         VNAWWKGIEGEMWDAVRCAITKINKKQKKNGTFSIDECGIFPP
PfNF54_120005700/1-319      VNAWWKGIEGEMWDAVRCAITKINKKQKKNGTFSIDECGIFPP
PfHB3_120006000/1-342       VNDWWKGIEGEMWGAVKCGIKTINKKQKKNGTYTGNECGVSPP
PfIT_120006100/1-328        VNAWWKGIEREMWDAVRCAITKINK.KNNNSIFNGDECGVSPP
Pf7G8_120005700/1-341       VNAWWKGIEGEMWDAVRCAITKIKK.QKKNGTFNGDECGVSPP
PfKH01_000031900/1-324      VNAWWKEIEKDMWGAVKCGIKTIKK.QKKNGTFNGDECGVSPP
PfGA01_120005700/1-325      VNAWWKGIEGEMWGAVRCAITKINK.KKKNGTFSIDECGIFPP
PfGB4_120005700/1-326       VNDWWKEIEGEMWDAVRCGITKINK.KNNNSIFNGNECGNSPS
PfKE01_120006100/1-325      VNDWWKEIEKDMWGAVKCGIKTIKK.QKKNGTFSIDECGVSPP
```

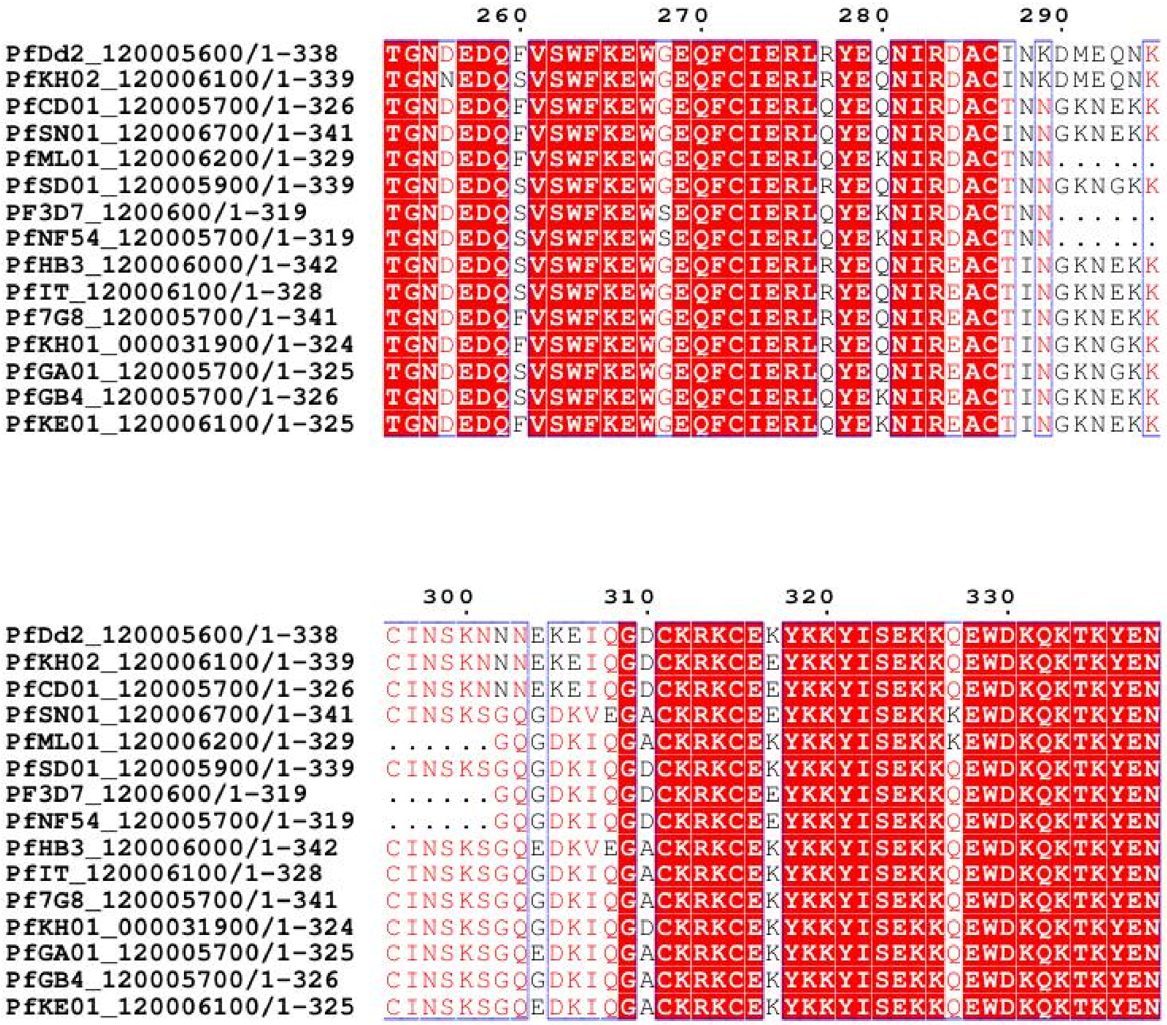

Supplementary Fig. 8. ESPript multiple sequence alignment of DBL3x domains of *P. falciparum* isolates.

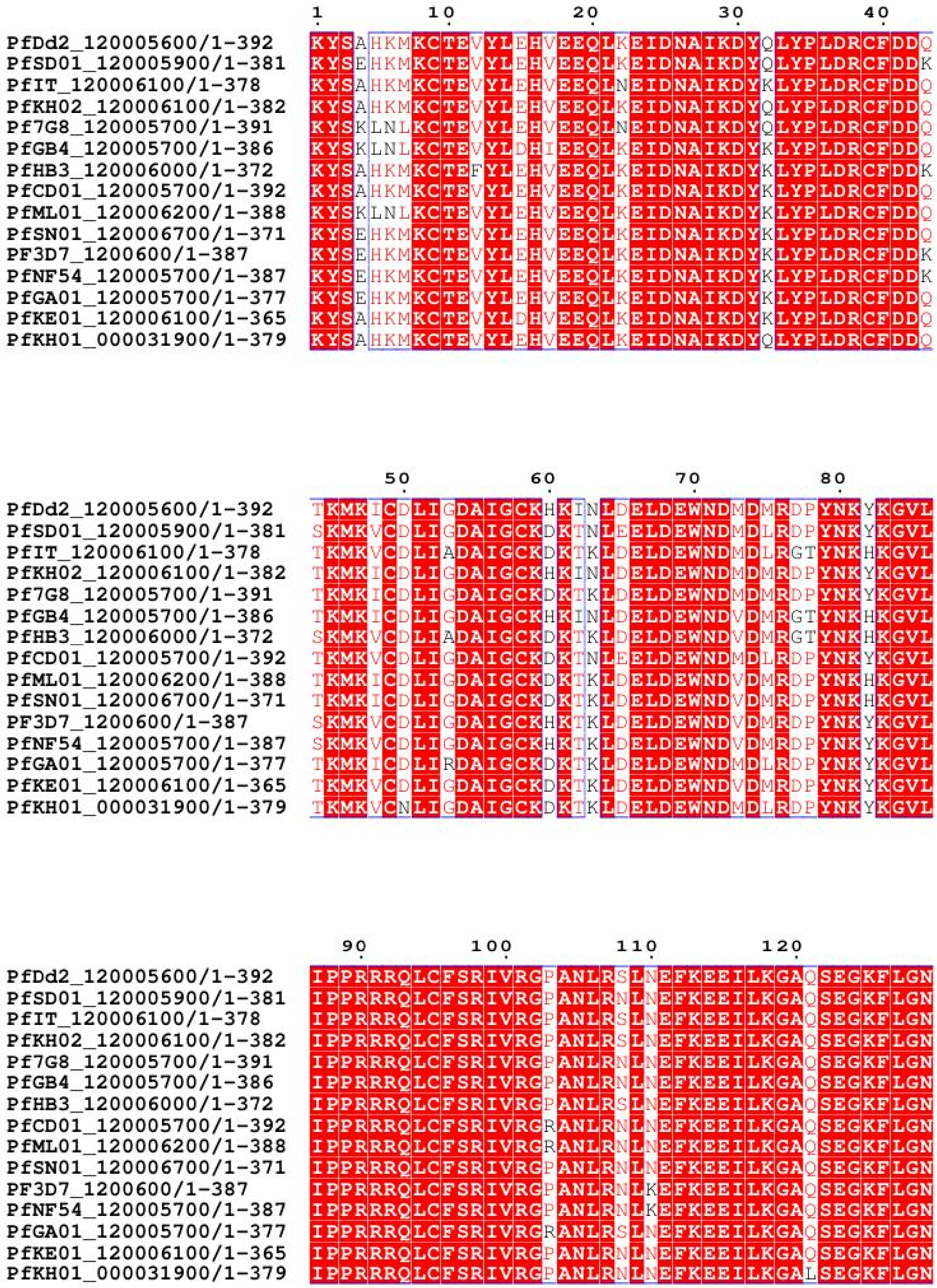

1 10 20 30 40
PfDd2_120005600/1-392 KYSAHKMKCTEVYLEHVEEQLKEIDNAIKDYQLYPLDRCFDDQ
PfSD01_120005900/1-381 KYSEHKMKCTEVYLEHVEEQLKEIDNAIKDYQLYPLDRCFDDK
PfIT_120006100/1-378 KYSAHKMKCTEVYLEHVEEQLNEIDNAIKDYKLYPLDRCFDDQ
PfKH02_120006100/1-382 KYSAHKMKCTEVYLEHVEEQLKEIDNAIKDYQLYPLDRCFDDQ
Pf7G8_120005700/1-391 KYSKLNLKCTEVYLEHVEEQLNEIDNAIKDYQLYPLDRCFDDQ
PfGB4_120005700/1-386 KYSKLNLKCTEVYLDHIEEQLKEIDNAIKDYKLYPLDRCFDDQ
PfHB3_120006000/1-372 KYSAHKMKCTEFYLEHVEEQLKEIDNAIKDYKLYPLDRCFDDK
PfCD01_120005700/1-392 KYSAHKMKCTEVYLEHVEEQLKEIDNAIKDYKLYPLDRCFDDQ
PfML01_120006200/1-388 KYSKLNLKCTEVYLEHVEEQLKEIDNAIKDYKLYPLDRCFDDQ
PfSN01_120006700/1-371 KYSEHKMKCTEVYLEHVEEQLKEIDNAIKDYKLYPLDRCFDDQ
PF3D7_1200600/1-387 KYSEHKMKCTEVYLEHVEEQLKEIDNAIKDYKLYPLDRCFDDK
PfNF54_120005700/1-387 KYSEHKMKCTEVYLEHVEEQLKEIDNAIKDYKLYPLDRCFDDK
PfGA01_120005700/1-377 KYSEHKMKCTEVYLEHVEEQLKEIDNAIKDYKLYPLDRCFDDQ
PfKE01_120006100/1-365 KYSAHKMKCTEVYLDHVEEQLKEIDNAIKDYKLYPLDRCFDDQ
PfKH01_000031900/1-379 KYSAHKMKCTEVYLEHVEEQLKEIDNAIKDYQLYPLDRCFDDQ
50 60 70 80
PfDd2_120005600/1-392 TKMKICDLIGDAIGCKHKINLDELDEWNDMDMRDPYNKYKGVL
PfSD01_120005900/1-381 SKMKVCDLIGDAIGCKDKTNLEELDEWNDMDLRDPYNKYKGVL
PfIT_120006100/1-378 TKMKVCDLIADAIGCKDKTKLDELDEWNDMDLRGTYNKHKGVL
PfKH02_120006100/1-382 TKMKICDLIGDAIGCKHKINLDELDEWNDMDMRDPYNKYKGVL
Pf7G8_120005700/1-391 TKMKICDLIGDAIGCKDKTKLDELDEWNDMDMRDPYNKYKGVL
PfGB4_120005700/1-386 TKMKICDLIGDAIGCKHKINLDELDEWNDVDMRGTYNKHKGVL
PfHB3_120006000/1-372 SKMKVCDLIADAIGCKDKTKLDELDEWNDVDMRGTYNKHKGVL
PfCD01_120005700/1-392 TKMKVCDLIGDAIGCKDKTNLEELDEWNDMDLRDPYNKYKGVL
PfML01_120006200/1-388 TKMKICDLIGDAIGCKDKTKLDELDEWNDVDMRDPYNKHKGVL
PfSN01_120006700/1-371 TKMKICDLIGDAIGCKDKTKLDELDEWNDVDMRDPYNKHKGVL
PF3D7_1200600/1-387 SKMKVCDLIGDAIGCKHKTKLDELDEWNDVDMRDPYNKYKGVL
PfNF54_120005700/1-387 SKMKVCDLIGDAIGCKHKTKLDELDEWNDVDMRDPYNKYKGVL
PfGA01_120005700/1-377 TKMKICDLIRDAIGCKDKTKLDELDEWNDMDLRDPYNKYKGVL
PfKE01_120006100/1-365 TKMKVCDLIGDAIGCKDKTKLDELDEWNDVDMRDPYNKYKGVL
PfKH01_000031900/1-379 TKMKVCNLIGDAIGCKDKTKLDELDEWNDMDLRDPYNKYKGVL
90 100 110 120
PfDd2_120005600/1-392 IPPRRRQLCFSRIVRGPANLRSLNEFKEEILKGAQSEGKFLGN
PfSD01_120005900/1-381 IPPRRRQLCFSRIVRGPANLRNLNEFKEEILKGAQSEGKFLGN
PfIT_120006100/1-378 IPPRRRQLCFSRIVRGPANLRSLNEFKEEILKGAQSEGKFLGN
PfKH02_120006100/1-382 IPPRRRQLCFSRIVRGPANLRSLNEFKEEILKGAQSEGKFLGN
Pf7G8_120005700/1-391 IPPRRRQLCFSRIVRGPANLRNLNEFKEEILKGAQSEGKFLGN
PfGB4_120005700/1-386 IPPRRRQLCFSRIVRGPANLRNLNEFKEEILKGAQSEGKFLGN
PfHB3_120006000/1-372 IPPRRRQLCFSRIVRGPANLRSLNEFKEEILKGAQSEGKFLGN
PfCD01_120005700/1-392 IPPRRRQLCFSRIVRGRANLRNLNEFKEEILKGAQSEGKFLGN
PfML01_120006200/1-388 IPPRRRQLCFSRIVRGRANLRNLNEFKEEILKGAQSEGKFLGN
PfSN01_120006700/1-371 IPPRRRQLCFSRIVRGPANLRNLNEFKEEILKGAQSEGKFLGN
PF3D7_1200600/1-387 IPPRRRQLCFSRIVRGPANLRNLKEFKEEILKGAQSEGKFLGN
PfNF54_120005700/1-387 IPPRRRQLCFSRIVRGPANLRNLKEFKEEILKGAQSEGKFLGN
PfGA01_120005700/1-377 IPPRRRQLCFSRIVRGRANLRSLNEFKEEILKGAQSEGKFLGN
PfKE01_120006100/1-365 IPPRRRQLCFSRIVRGPANLRNLNEFKEEILKGAQSEGKFLGN
PfKH01_000031900/1-379 IPPRRRQLCFSRIVRGPANLRNLNEFKEEILKGALSEGKFLGN

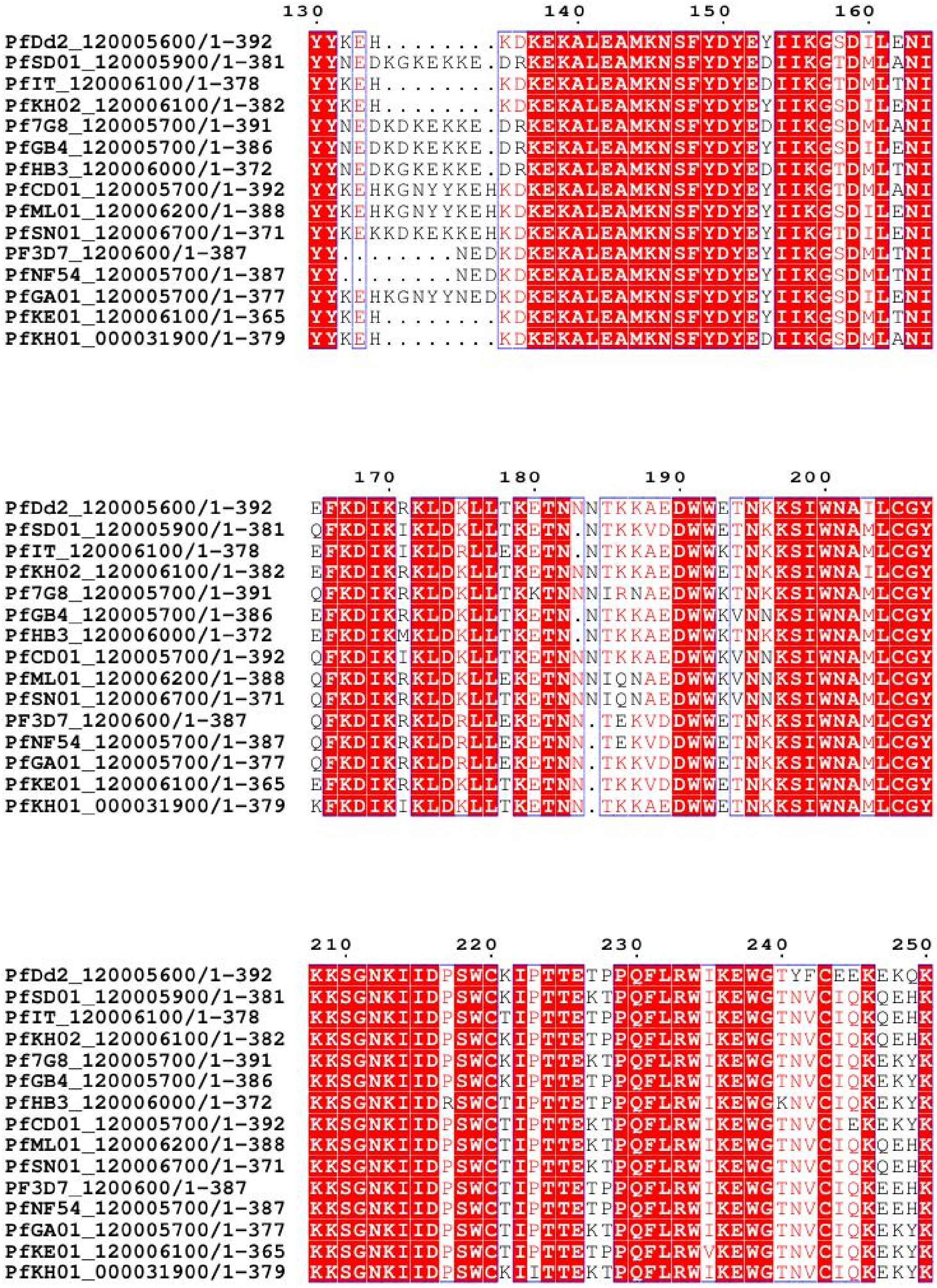
130 140 150 160
PfDd2_120005600/1-392 YYKEH........KDKEKALEAMKNSFYDYEYIIKGSDILENI
PfSD01_120005900/1-381 YYNEDKGKEKKE.DRKEKALEAMKNSFYDYEDIIKGTDMLANI
PfIT_120006100/1-378 YYKEH........KDKEKALEAMKNSFYDYEDIIKGTDMLTNI
PfKH02_120006100/1-382 YYKEH........KDKEKALEAMKNSFYDYEYIIKGSDILENI
Pf7G8_120005700/1-391 YYNEDKDKEKKE.DRKEKALEAMKNSFYDYEDIIKGSDMLANI
PfGB4_120005700/1-386 YYNEDKDKEKKE.DRKEKALEAMKNSFYDYEYIIKGSDILENI
PfHB3_120006000/1-372 YYNEDKGKEKKE.DRKEKALEAMKNSFYDYEDIIKGTDMLTNI
PfCD01_120005700/1-392 YYKEHKGNYYKEHKDKEKALEAMKNSFYDYEYIIKGTDMLANI
PfML01_120006200/1-388 YYKEHKGNYYKEHKDKEKALEAMKNSFYDYEYIIKGSDILENI
PfSN01_120006700/1-371 YYKEKKDKEKKEHKDKEKALEAMKNSFYDYEYIIKGTDILENI
PF3D7_1200600/1-387 YY........NEDKDKEKALEAMKNSFYDYEYIIKGSDMLTNI
PfNF54_120005700/1-387 YY........NEDKDKEKALEAMKNSFYDYEYIIKGSDMLTNI
PfGA01_120005700/1-377 YYKEHKGNYYNEDKDKEKALEAMKNSFYDYEYIIKGSDILENI
PfKE01_120006100/1-365 YYKEH........KDKEKALEAMKNSFYDYEYIIKGSDMLTNI
PfKH01_000031900/1-379 YYKEH........KDKEKALEAMKNSFYDYEDIIKGSDMLANI
170 180 190 200
PfDd2_120005600/1-392 EFKDIKRKLDKLLTKETNNNTKKAEDWWETNKKSIWNAILCGY
PfSD01_120005900/1-381 QFKDIKIKLDKLLTKETN.NTKKVDDWWETNKKSIWNAMLCGY
PfIT_120006100/1-378 EFKDIKIKLDRLLEKETN.NTKKAEDWWKTNKKSIWNAMLCGY
PfKH02_120006100/1-382 EFKDIKRKLDKLLTKETNNNTKKAEDWWETNKKSIWNAILCGY
Pf7G8_120005700/1-391 QFKDIKRKLDKLLTKKTNNNIRNAEDWWKTNKKSIWNAMLCGY
PfGB4_120005700/1-386 EFKDIKRKLDKLLTKETN.NTKKAEDWWKVNNKSIWNAMLCGY
PfHB3_120006000/1-372 EFKDIKMKLDKLLTKETN.NTKKAEDWWKTNKKSIWNAMLCGY
PfCD01_120005700/1-392 QFKDIKIKLDKLLTKETNNNTKKAEDWWKVNNKSIWNAMLCGY
PfML01_120006200/1-388 QFKDIKRKLDKLLEKETNNNIQNAEDWWKVNNKSIWNAMLCGY
PfSN01_120006700/1-371 QFKDIKRKLDKLLTKETNNNIQNAEDWWKVNNKSIWNAMLCGY
PF3D7_1200600/1-387 QFKDIKRKLDRLLEKETNN.TEKVDDWWETNKKSIWNAMLCGY
PfNF54_120005700/1-387 QFKDIKRKLDRLLEKETNN.TEKVDDWWETNKKSIWNAMLCGY
PfGA01_120005700/1-377 QFKDIKRKLDRLLEKETNN.TKKVDDWWETNKKSIWNAMLCGY
PfKE01_120006100/1-365 EFKDIKRKLDKLLTKETNN.TKKVDDWWETNKKSIWNAMLCGY
PfKH01_000031900/1-379 KFKDIKIKLDKLLTKETNN.TKKAEDWWETNKKSIWNAMLCGY
210 220 230 240 250
PfDd2_120005600/1-392 KKSGNKIIDPSWCKIPTTETPPQFLRWIKEWGTYFCEEKEKQK
PfSD01_120005900/1-381 KKSGNKIIDPSWCKIPTTEKTPQFLRWIKEWGTNVCIQKQEHK
PfIT_120006100/1-378 KKSGNKIIDPSWCTIPTTETPPQFLRWIKEWGTNVCIQKQEHK
PfKH02_120006100/1-382 KKSGNKIIDPSWCKIPTTETPPQFLRWIKEWGTNVCIQKQEHK
Pf7G8_120005700/1-391 KKSGNKIIDPSWCKIPTTEKTPQFLRWIKEWGTNVCIQKEKYK
PfGB4_120005700/1-386 KKSGNKIIDPSWCKIPTTETPPQFLRWIKEWGTNVCIQKEKYK
PfHB3_120006000/1-372 KKSGNKIIDRSWCTIPTTETPPQFLRWIKEWGKNVCIQKEKYK
PfCD01_120005700/1-392 KKSGNKIIDPSWCTIPTTEKTPQFLRWIKEWGTNVCIEKEKYK
PfML01_120006200/1-388 KKSGNKIIDPSWCTIPTTEKTPQFLRWIKEWGTNVCIQKQEHK
PfSN01_120006700/1-371 KKSGNKIIDPSWCTIPTTEKTPQFLRWIKEWGTNVCIQKQEHK
PF3D7_1200600/1-387 KKSGNKIIDPSWCTIPTTETPPQFLRWIKEWGTNVCIQKEEHK
PfNF54_120005700/1-387 KKSGNKIIDPSWCTIPTTETPPQFLRWIKEWGTNVCIQKEEHK
PfGA01_120005700/1-377 KKSGNKIIDPSWCTIPTTEKTPQFLRWIKEWGTNVCIQKEKYK
PfKE01_120006100/1-365 KKSGNKIIDPSWCTIPTTETPPQFLRWVKEWGTNVCIQKEKYK
PfKH01_000031900/1-379 KKSGNKIIDPSWCKIITTEKTPQFLRWIKEWGTNVCIQKEKYK

```
                                           260              270              280              290
PfDd2_120005600/1-392      NIVNIKCTDSDHIIGKSIKASEKLECKLATGNYEQWNTNRLKE
PfSD01_120005900/1-381     EYVKSKCSNVTN...LGAQASESNNCIPEIRKYQEWSRKRSIQ
PfIT_120006100/1-378       EYVKSKCSNVTNL...GAQASESNNCTSEIKKYQEWSRKRSIQ
PfKH02_120006100/1-382     EYVKSKCSNVTNI.DLSLQASESKNCTSEIRKYQEWSRKRSIQ
Pf7G8_120005700/1-391      QNVKLECSNVTNI.DLSLQASESTKCIPEIRKYQEWSRKRSIQ
PfGB4_120005700/1-386      QNVKLECSNVSNI.DLSLQALESKNCTSEIRKYQEWSRKRSIQ
PfHB3_120006000/1-372      QNVKSECSNVSNI.DLDPQASESNNCTSEIRKYQELIRKRSIQ
PfCD01_120005700/1-392     EYVKSKCSNVTNI.DLDPQASESKNCTSEIRKYQEWSRNRYVQ
PfML01_120006200/1-388     EYVKSKCSNVTNN.NLGSQESESKNCTSEIRKYQEWSRKRSIQ
PfSN01_120006700/1-371     EYVKSKCSNVTNI.DLDPQASESKNCTSEIRKYQEWSRKRSIQ
PF3D7_1200600/1-387        EYVKSKCSNVT...NLGAQESESKNCTSEIKKYQEWSRKRSIQ
PfNF54_120005700/1-387     EYVKSKCSNVT...NLGAQESESKNCTSEIKKYQEWSRKRSIQ
PfGA01_120005700/1-377     QNVKSECSNVPNN.NLGSQESESNNCTSEIRKYQEWSRKRSIQ
PfKE01_120006100/1-365     QNVKSKCSNVPNN.NLGSQESESKNCTSEIRKYQEWSRKRSIQ
PfKH01_000031900/1-379     KNVKSKCSNVPNN.NLGSQESESKNCTSEIRKYQEWSRKRSIQ
```

```
                                 300                   310         320
PfDd2_120005600/1-392      WKLLSQQYKTNKESN..........KDIDMSELSAEQYLKKHCT
PfSD01_120005900/1-381     WETISKRYKR...MDILK....DVK.....EPDANEYLKEYCS
PfIT_120006100/1-378       WETISKRYKKYKRMDILKD....VKEP.....DANTYLREHCS
PfKH02_120006100/1-382     WEAISERYRKYKGMDVLKN....VKEP.....DANEYLKEYCS
Pf7G8_120005700/1-391      WEAISERYRKYKRMDEFKNVFNNANEPN.....ANEYLKEYCS
PfGB4_120005700/1-386      WEAISERYRKYKGMDEFKNV....KEPD.....ANEYLKEHCS
PfHB3_120006000/1-372      WEAISERYRKYKGMDVLKNV....KEPD.....ANEYLKEHCS
PfCD01_120005700/1-392     WEAISERYKKYKRMDEFKNTFKNIKEHD.....ANEYLKEHCS
PfML01_120006200/1-388     WEAISERYKKYKGMDIL....KDVKEPD.....ANTYLKEHCS
PfSN01_120006700/1-371     WEAISERYK...RMDIL....KDVKESD.....ANEYLKEHCS
PF3D7_1200600/1-387        WEAISEGYKKYKGMDEFKNTFKNIKEPDANEPNANEYLKKHCS
PfNF54_120005700/1-387     WEAISEGYKKYKGMDEFKNTFKNIKEPDANEPNANEYLKKHCS
PfGA01_120005700/1-377     WEAISERYKKYKGMDEFKNTFKNIKEPD.....ANTYLREHCS
PfKE01_120006100/1-365     WEAISERYKKYKRMDEF....KNVKEPD.....ANEYLKEHCS
PfKH01_000031900/1-379     WEAISEGYKKYKGKDEF....KNVKEPD.....ANTYLKEHCS
```

```
                           330        340        350        360        370
PfDd2_120005600/1-392      GCSCRFSDMDELYIKTLKNENLHEEILKNAQIPIYFEEVYHRF
PfSD01_120005900/1-381     KCPCGFNDMQEITNDNDKVEEIFNRIIEKVKIPAELEDVIYRI
PfIT_120006100/1-378       KCPCGFNDMEEMNNNEDNEKEAFKQIKEQVKIPAELEDVIYRI
PfKH02_120006100/1-382     KCPCGYNDMEEMNNNEDNEKEGFKQIKEQVDIPAELEDVIYRI
Pf7G8_120005700/1-391      KCPCGFNDMEEITNDTENKQDIFKQIIEKVKIPAELEDVIYRL
PfGB4_120005700/1-386      KCPCGFNDMKEIANDTENKQDIFKQIKEQVDIPAELEDVIYRL
PfHB3_120006000/1-372      KCPCGYNDMQEIANDTENKQDIFKQIKEQVDIPAELEDVI...
PfCD01_120005700/1-392     KCPCGFNDMQEITKYTNIGNEAFKQIKEQVDIPAELEDVIYRL
PfML01_120006200/1-388     KCPCGFNDMKEISNNEDNEKETFNRIIEKVKIPAELEDVIYRL
PfSN01_120006700/1-371     KCPCGFNDMKEIANDTENKQDIFKQIKEQVDIPAELEDVI...
PF3D7_1200600/1-387        KCPCGFNDMQEITKYTNIGNEAFKQIKEQVDIPAELEDVIYRL
PfNF54_120005700/1-387     KCPCGFNDMQEITKYTNIGNEAFKQIKEQVDIPAELEDVIYRL
PfGA01_120005700/1-377     KCPCGFNDMKEIANDTENKQDIFNRIKEQVDIPAELEDVI...
PfKE01_120006100/1-365     KCPCGYNDMEEIANDTENKQDIFKQIIEKVKIPAELEDVI...
PfKH01_000031900/1-379     KCPCGFNDMEEIANDTENKQDIFKQIKEQVDIPDELEDVIYRI
```

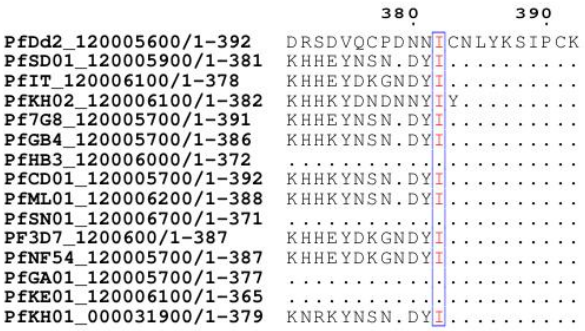

Supplementary Fig. 9. ESPript multiple sequence alignment of DBL5ε domains of *P. falciparum* isolates.

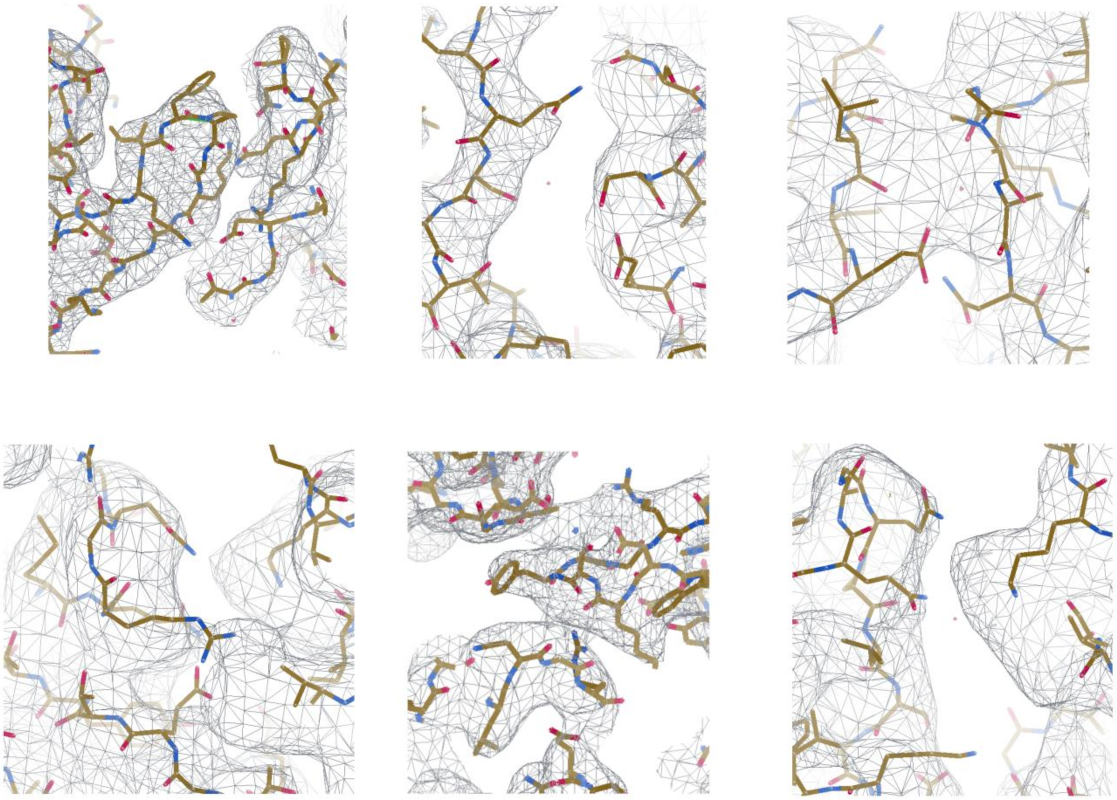

Supplementary Fig. 10. Representative fit of the model side chains of the map

Data Collection and Processing	IgM-VAR2CSA complex EMD-34399, PDB 8GZN
Voltage	300(keV)
Microscope	FEI Titan Krios
Detector	falcon3
Total Electron exposure (e/A^2)	51.2
Exposure Rate (e/Å^2/S)	0.98
Exposure Rate (e/Å^2/S)	0.81
Number of Frames collected per micrograph	50
Data collection Software	EPU
Defocus range (um)	-0.8 to -2.2
Pixel size	1.10
Micrographs used	15668
Symmetry imposed	C1
Final particle in Refine	371049
Resolution of map at 0.143 FSC (masked)	3.6
Map Sharpening	None
Initial Models used	6KXS, 7NNH, 7B52
Refinement software	PHENIX
CC mask	0.82
CC Box	0.82
CC Peaks	0.64
CC Volume	0.81
Model composition (non-hydrogen)	35402
Protein Residues	4409
Bonds RMSD	
Length (Å)	0.003
Angles	0.693
B factor (Å$^{2)}$	244
Validation	
MolProbity score	2.24
Clashscore	15.38
Ramachandran Plot (%)	
Favored	90.53
Allowed	9.45
Outliers	0.02
Rama-Z	
Whole (N= 4327)	-1.11
Helix (N = 1389)	0.69
Sheet (N= 753)	-0.47
Loop (N=2203)	-1.82
Cbeta outliers	0.00
CaBLAM outliers	5.80

Supplementary Table 1 Cryo-EM data collection, processing and real space refinement validation statistics
