# Supplementary material for: Cryo-electron microscopy of IgM-VAR2CSA complex reveals IgM inhibits binding of Plasmodium falciparum to Chondroitin Sulfate A
Source: Nat Commun. 2023 Oct 12;14:6391. doi: 10.1038/s41467-023-41838-x (PMC10570280; doi:10.1038/s41467-023-41838-x)
Supplement: Supplementary file 3 — Reporting Summary [file 41467_2023_41838_MOESM3_ESM.pdf]

nature portfolio

Corresponding author(s): Reeetesh Raj Akhouri

Last updated by author(s): Sep 7, 2023

Reporting Summary

Nature Portfolio wishes to improve the reproducibility of the work that we publish. This form provides structure for consistency and transparency in reporting. For further information on Nature Portfolio policies, see our Editorial Policies and the Editorial Policy Checklist.

Statistics

For all statistical analyses, confirm that the following items are present in the figure legend, table legend, main text, or Methods section.

n/a	Confirmed	
☐	☒	The exact sample size (*n*) for each experimental group/condition, given as a discrete number and unit of measurement
☐	☒	A statement on whether measurements were taken from distinct samples or whether the same sample was measured repeatedly
☐	☒	The statistical test(s) used AND whether they are one- or two-sided<br>*Only common tests should be described solely by name; describe more complex techniques in the Methods section.*
☒	☐	A description of all covariates tested
☒	☐	A description of any assumptions or corrections, such as tests of normality and adjustment for multiple comparisons
☐	☒	A full description of the statistical parameters including central tendency (e.g. means) or other basic estimates (e.g. regression coefficient) AND variation (e.g. standard deviation) or associated estimates of uncertainty (e.g. confidence intervals)
☐	☒	For null hypothesis testing, the test statistic (e.g. *F*, *t*, *r*) with confidence intervals, effect sizes, degrees of freedom and *P* value noted<br>*Give P values as exact values whenever suitable.*
☒	☐	For Bayesian analysis, information on the choice of priors and Markov chain Monte Carlo settings
☒	☐	For hierarchical and complex designs, identification of the appropriate level for tests and full reporting of outcomes
☒	☐	Estimates of effect sizes (e.g. Cohen's *d*, Pearson's *r*), indicating how they were calculated

Our web collection on statistics for biologists contains articles on many of the points above.

Software and code

Policy information about availability of computer code

Data collection	Cryo-EM data collection-EPU (Thermofisher), BD FACS Diva, Confocal-LASX, Unicorn V-AKTA explorer, chemiluminescence-Amersham Imager AI-600
Data analysis	Image processing-RELION 3.1, COOT 0.98, PHENIX V1.20.1, Chimera, ChimeraX, PyMOL2.5.5, MS EXCEL workbook, FlowJO v10.9, CLustal Omega, Weblogo 3, ESPript3

For manuscripts utilizing custom algorithms or software that are central to the research but not yet described in published literature, software must be made available to editors and reviewers. We strongly encourage code deposition in a community repository (e.g. GitHub). See the Nature Portfolio guidelines for submitting code & software for further information.

Data

Policy information about availability of data

All manuscripts must include a data availability statement. This statement should provide the following information, where applicable:

- Accession codes, unique identifiers, or web links for publicly available datasets
- A description of any restrictions on data availability
- For clinical datasets or third party data, please ensure that the statement adheres to our policy

The Cryo-EM map and atomic coordinates of the IgM-VAR2CSA complex has been deposited in the EMDB and PDB with an accession code EMD-34399 and 8GZN respectively. All constructs used for protein expression in this study are available upon request.

Research involving human participants, their data, or biological material

Policy information about studies with human participants or human data. See also policy information about sex, gender (identity/presentation), and sexual orientation and race, ethnicity and racism.

Reporting on sex and gender	N/A
Reporting on race, ethnicity, or other socially relevant groupings	N/A
Population characteristics	N/A
Recruitment	N/A
Ethics oversight	N/A

Note that full information on the approval of the study protocol must also be provided in the manuscript.

Field-specific reporting

Please select the one below that is the best fit for your research. If you are not sure, read the appropriate sections before making your selection.

☒ Life sciences ☐ Behavioural & social sciences ☐ Ecological, evolutionary & environmental sciences

For a reference copy of the document with all sections, see nature.com/documents/nr-reporting-summary-flat.pdf

Life sciences study design

All studies must disclose on these points even when the disclosure is negative.

Sample size	The experiment was performed 3 times in duplicates and 5 fields were counted per spot and represented as a point in the plot. Sample size was based on the number of IEs bound to CSA. As total pRBCs bound in experiments were above 10,000 per experiment, this number is sufficient enough to remove bias and perform statistical analysis
Data exclusions	None
Replication	The experiments were performed three times and all experiments were successful
Randomization	All experiments ere successful. We have coated CSA and added IEs in the petriplate at different places in a randomized manner to avoid washing and binding bias. However, this is not computer generated and hence we do not consider randomization. We label spots and source of IEs and therefore it is not random.
Blinding	Investigators were blinded

Reporting for specific materials, systems and methods

We require information from authors about some types of materials, experimental systems and methods used in many studies. Here, indicate whether each material, system or method listed is relevant to your study. If you are not sure if a list item applies to your research, read the appropriate section before selecting a response.

Materials & experimental systems

n/a	Involved in the study	
☐	☒	Antibodies
☐	☒	Eukaryotic cell lines
☒	☐	Palaeontology and archaeology
☐	☒	Animals and other organisms
☒	☐	Clinical data
☒	☐	Dual use research of concern
☐	☐	Plants

Methods

n/a	Involved in the study	
☒	☐	ChIP-seq
☐	☒	Flow cytometry
☒	☐	MRI-based neuroimaging

Antibodies

Antibodies used	IgM (jackson Immunoresearch Cat# 009-000-012),<br>Anti-VAR2CSA antibody- custom produced in animal house,<br>donkey-anti-rabbit Alexa Fluor 594 (Cat# A-21207, Thermofisher Scientific),

J Chain Polyclonal antibody (Cat# PA583707, Thermofisher Scientific),
F(ab')2-Goat anti human IgM Heacy Chain secondary antibody (Cat#A24484, Invitrogen),
donkey anti rabbit HRP (NA934-1ml, Cytiva),
sheep anti-mouse HRP (NA931-1ml, Cytiva),
PfHSP70 (Cat# SPC186, StressMarq),
Penta His antibody (Cat# 34660, Qiagen)

Validation

IgM for structure,
anti-VAR2CSA antibodies were validated using western blot analysis where only anti-rabbit HRP alone was used as control. Donkey anti-rabbit Alexa Fluor 594 citation from Thermofisher website: Incomplete activation of Alyref and Gabpb1 leads to preimplantation arrest in cloned mouse embryos. Life Sci Alliance (2023);
J chain Polyclonal antibody- The antibody was verified by relative expression to ensure that the antibody binds to the antigen stated;
anti-mouse HRP NA931-1m citation-Two forms of Opa1 cooperate to complete fusion of the mitochondrial inner-membrane.
Yifan Ge et al.eLife, 9 (2020-01-11)
PfHSP70- Our previous studies Goel S et al Nature medicine 2015 has shown the validation of the antibody
Penta His antibody-validated in the website by detection of 6X His tagged proteins with antihis antibodies: DHFR, Thioredoxin, TNF-a, chaperonin

Eukaryotic cell lines

Policy information about cell lines and Sex and Gender in Research

Cell line source(s)	Plasmodium falciparum strain CS2-MR4, Drosophila S2 cells-Thermofisher
Authentication	CS2 selection on CSA, S2-Blasticidin drug sensitivity
Mycoplasma contamination	Negative
Commonly misidentified lines (See ICLAC register)	No misidentified cell lines were used in the study

Animals and other research organisms

Policy information about studies involving animals; ARRIVE guidelines recommended for reporting animal research, and Sex and Gender in Research

Laboratory animals	New Zealand White Rabbit for production of anti-VAR2CSA antibodies, 12 weeks old
Wild animals	None
Reporting on sex	None
Field-collected samples	None
Ethics oversight	Study was approved by IAEC, SRM Chennai, India

Note that full information on the approval of the study protocol must also be provided in the manuscript.

Flow Cytometry

Plots

Confirm that:

- [x] The axis labels state the marker and fluorochrome used (e.g. CD4-FITC).
- [x] The axis scales are clearly visible. Include numbers along axes only for bottom left plot of group (a 'group' is an analysis of identical markers).
- [ ] All plots are contour plots with outliers or pseudocolor plots.
- [x] A numerical value for number of cells or percentage (with statistics) is provided.

Methodology

Sample preparation

The anti-VAR2CSA antibody was pre-absorbed with uninfected red blood cells (RBCs) overnight at 4°C. The CS2 culture at 10% parasitemia was washed thrice with PBS and incubated with pre-absorbed anti-VAR2CSA antibody and non-immune IgG from rabbit at a concentration of 100 µg/mL in PBS + 2% BSA for 1 h at RT. The parasites were washed four times with PBS and incubated with 1:100 diluted anti-rabbit Alexa Fluor 594 and 2.5 µg/mL ethidium bromide for 1 h at RT. As a negative control, the parasites were only incubated with anti-rabbit Alexa Fluor 594 (1:100, Cat# A-21207, Thermofisher Scientific)and 2.5 µg/mL ethidium bromide. The parasites were washed four times with PBS and finally resuspended in PBS for FACS analysis using FACS-Celesta (Becton, Dickinson and Company, Franklin Lakes, NJ). For acquisition, unstained cells were used to gate RBCs, while ethidium bromide staining was used to gate IEs and only secondary antibody was used to adjust the gate for the

	positive staining with anti-VAR2CSA antibody. The VAR2CSA positive cells were tested from 10,000 IEs, and the data were analyzed using FlowJo v10.9 software, where expression of VAR2CSA was compared with CS2 grown in albumax and plasma containing media for 10,000 IEs
Instrument	BD FACS Celesta
Software	BD FACS Diva, FlowJo V10.9
Cell population abundance	10,000 IEs were accquired from 10% parasitemia culture
Gating strategy	First gating: RBC were gated (P1), second gating:IEs were gated (P2)

☒ Tick this box to confirm that a figure exemplifying the gating strategy is provided in the Supplementary Information.
